# Efficacy and safety of hetrombopag in the treatment of chemotherapy-related thrombocytopenia in solid tumors: a retrospective study

**DOI:** 10.3389/fonc.2025.1670029

**Published:** 2025-10-28

**Authors:** Yuan Yuan, Qiang Tong, Jia-hui Liu, Ye Kang

**Affiliations:** Department of Pharmacy, General Hospital of Northern Theater Command, Shenyang, China

**Keywords:** hetrombopag, solid tumors, thrombopoietin-receptor agonist (TPO-RA), chemotherapy-induced thrombocytopenia (CIT), recombinant human thrombopoietin (rhTPO)

## Abstract

**Objective:**

This study aims to evaluate the efficacy and safety of hetrombopag in the management of chemotherapy-induced thrombocytopenia (CIT) among patients with solid tumors, utilizing a retrospective cohort study design.

**Methods:**

The study population comprised patients experiencing CIT due to chemotherapy for solid tumors, who received treatment at the General Hospital of Northern Theater Command from January 2023 to December 2024. Participants were categorized into four distinct cohorts based on their treatment regimens: the recombinant human thrombopoietin (rhTPO) monotherapy group, the hetrombopag monotherapy group, the combination therapy group (hetrombopag with rhTPO), and the recombinant human interleukin-11 (rhIL-11) monotherapy group. The primary outcomes evaluated included treatment response rate, alterations in platelet count (PLT), time to PLT recovery, differences in PLT counts pre- and post-treatment; secondary outcome measured encompassed rates of platelet transfusion, and incidence of adverse events (AEs).

**Results:**

The study included a total of 187 patients, distributed as follows: 64 in the rhTPO group, 37 in the hetrombopag group, 36 in the combination therapy group, and 50 in the rhIL-11 group. The hetrombopag + rhTPO group exhibited a significantly higher treatment response rate (P<0.05) compared to the other three groups. Furthermore, this group showed superior PLT levels on days 7 and 14, a greater increment in PLT post-treatment, and the shortest median time to PLT recovery to ≥100×10^9^/L (P<0.05). Hetrombopag monotherapy demonstrated non-inferior PLT dynamics and treatment response rates compared to rhTPO/rhIL-11 (P>0.05). The four groups exhibited comparable PLT transfusion rates and a AEs incidence (P>0.05).

**Conclusion:**

The combination of hetrombopag and rhTPO therapy exhibits superior efficacy compared to monotherapy in the treatment of CIT in patients with solid tumors. This combination therapy is associated with rapid elevation of platelet counts and a shortened recovery period, while maintaining a favorable safety profile. Furthermore, hetrombopag monotherapy has shown efficacy comparable to that of rhTPO and recombinant human interleukin-11 (rhIL-11), thereby supporting its recommendation for clinical use.

## Introduction

Chemotherapy-induced thrombocytopenia (CIT) is a prevalent hematologic toxicity resulting from chemotherapy agents that inhibit megakaryocyte production in the bone marrow, leading to a peripheral blood platelet count of less than 100×10^9^/L (1). CIT not only elevates the risk of hemorrhage but also necessitates chemotherapy dose reductions, delays in treatment, or even discontinuation, thereby significantly compromising patient survival outcomes (2). Current management strategies for CIT predominantly involve the use of platelet transfusions and thrombopoiesis-stimulating agents, with recombinant human thrombopoietin (rhTPO) and recombinant human interleukin-11 (rhIL-11) being the primary agents employed. However, these agents have significant limitations. RhTPO can lead to the development of neutralizing antibodies, which reduce platelet responsiveness and diminish therapeutic efficacy, while rhIL-11 is frequently associated with adverse events such as fever, edema, and arrhythmias (3). Additionally, the injectable nature of these treatments results in suboptimal patient compliance. In recent years, novel non-peptide oral thrombopoietin receptor agonists (TPO-RAs), such as hetrombopag, have shown promising therapeutic potential. These agents specifically bind to the transmembrane domain of thrombopoietin receptors, activating downstream signaling pathways including STAT, PI3K, and ERK, thereby promoting megakaryocyte differentiation and maturation. This mechanism has resulted in an 80% response rate in patients with immune thrombocytopenia (ITP) (4). However, existing research has predominantly concentrated on hematologic malignancies, with limited clinical evidence specifically addressing CIT. Although the Chinese Expert Consensus on Diagnosis and Management of Drug-Related Thrombocytopenia in Oncology (2023 Edition) and the CSCO Guidelines for the Management of Thrombocytopenia Induced by Antitumor Therapy (2024 Edition) include hetrombopag as a second-line therapeutic option, these recommendations are largely based on expert consensus rather than robust evidence-based data (5, 6). To bridge this gap, the present study seeks to systematically evaluate the platelet response rate and treatment-related adverse events associated with hetrombopag in CIT patients through a real-world retrospective cohort analysis. The findings are expected to provide critical evidence to optimize CIT management strategies and inform clinical decision-making.

## Materials and methods

### Study population and inclusion/exclusion criteria

This study included patients diagnosed with CIT associated with solid tumors, who were admitted to the General Hospital of Northern Theater Command from January 2023 to December 2024. The study employed a retrospective cohort design, utilizing data extracted from the Electronic Medical Record System (EMRS) of General Hospital of Northern Theater Command. Through a thorough examination of electronic medical records and pertinent laboratory test results, the incidence of outcome events was systematically monitored and analyzed.

The inclusion criteria were as follows: (1) age between 18 and 80 years; (2) histopathologically confirmed malignant tumor; (3) meeting the diagnostic criteria for CIT as outlined in the “Chinese Expert Consensus on the Diagnosis and Treatment of Cancer Drug-related Thrombocytopenia(2019 Edition)” (7)(platelet count <100×10^9^/L, excluding other etiologies); (4) Eastern Cooperative Oncology Group (ECOG) performance status of ≤2.

The exclusion criteria encompassed: (1) thrombocytopenia resulting from non-chemotherapy-related factors, such as immune thrombocytopenia or myelodysplastic syndrome; (2) uncontrolled infections, significant organ dysfunction, or coagulation abnormalities; (3) concurrent use of medications that affect platelet function, including anticoagulants and nonsteroidal anti-inflammatory drugs; and (4) known allergies to the study drugs.

The study protocol received approval from the Ethics Committee of the General Hospital of Northern Theater Command (approval number: 2025-Y-129), and the requirement for patient informed consent was waived by the committee due to the retrospective nature of the analysis and the absence of any intervention.

### Study grouping and treatment regimen

In this study, eligible patients who met the inclusion and exclusion criteria were categorized into four groups based on their treatment regimens: the rhTPO group received a daily subcutaneous injection of rhTPO at a dosage of 15,000 units, manufactured by Shenyang Sanyou Pharmaceutical Co., Ltd. (National Drug Approval Number S20050048); the hetrombopag group was administered an oral dose of hetrombopag at 5 mg per day, produced by Jiangsu Hengrui Pharmaceutical Co., Ltd. (National Drug Approval Number H20210021); the combined therapy group received both oral hetrombopag (5 mg per dose) and subcutaneous rhTPO injection (15,000 units per dose) once daily; the rhIL-11 group was treated with a daily subcutaneous injection of recombinant human interleukin-11 powder, at a dosage of 3 mg, supplied by Qilu Pharmaceutical Co., Ltd. (National Drug Approval Number S20053046). All four treatment cohorts underwent a continuous 14-day therapy regimen without any dose modifications throughout the observation period.

### Outcome measures

#### Primary outcome measures

1. Treatment efficacy rate;

The treatment efficacy rate was defined by meeting at least one of the following criteria: (1) a platelet count (PLT) of ≥100×10^9^/L; (2) an increase in PLT of ≥50×10^9^/L from baseline; or (3) PLT recovery to≥2 times the baseline level.

2. Assessment of absolute PLT values at baseline and at post-treatment intervals on Days 3, 7, and 14;

3. The number of days required for PLT recovery to ≥100×10^9^/L and the change in platelet count (ΔPLT) before and after treatment.

#### Secondary outcome measures

1. The proportion of patients receiving platelet transfusions across four treatment groups;

Transfusion criteria defined as treatment ineffectiveness, administered at a daily dose of 1 unit (equivalent to 2.5×10¹¹ PLT).

2. Incidence of Adverse Events (AEs): including the most common reactions observed in two Phase I trials of hetrombopag, elevated transaminases, hyperbilirubinemia, fatigue, and headache. Adverse event occurrences were determined through comprehensive analysis of electronic medical record progress notes and corroborating laboratory test results.

### Statistical analysis methods

Data analysis was conducted using SPSS version 29.0 (IBM Corp., Armonk, NY, USA), and data visualization was performed with GraphPad Prism version 10.0 (GraphPad Software, San Diego, CA, USA). For continuous variables, normally distributed data were presented as mean ± standard deviation (SD) and analyzed using one-way analysis of variance (ANOVA). Non-normally distributed data were reported as medians with interquartile ranges [P25, P75] and analyzed using the nonparametric Kruskal-Wallis test. Statistical significance was defined as a P-value of less than 0.05. Categorical variables were presented as percentages and analyzed using either the Chi-square test or Fisher’s exact test, with a P-value of less than 0.05 considered significant.

## Result

### Baseline characteristics

The study enrolled a total of 187 patients, consisting of 100 males (53.5%) and 87 females (46.5%). The median age of the participants was 60 years, with an interquartile range of 52 to 66 years. The primary tumor types included gastrointestinal malignancies (34.8%), gynecological malignancies (34.8%), and lung cancer (19.2%), together comprising 88.8% of the cohort. No significant differences were detected in demographic and clinical characteristics across the four treatment groups (P > 0.05), as detailed in **Table 1**.

**Table 1:** Baseline characteristics across treatment groups.

Characteristic	rhTPO Group (n=64)	Hetrombopag group (n=37)	Combination group (n=36)	rhIL-11 Group (n=50)	F/χ²	P-value
Gender, n (%)					2.598	0.458
Male	36 (56.3)	22 (59.5)	20 (55.6)	22 (44.0)		
Female	28 (43.7)	15 (40.5)	16 (44.4)	28 (56.0)		
Age (years)	59.72 ± 9.85	58.05 ± 9.98	54.69 ± 11.72	58.68 ± 9.62	1.928	0.127
BMI (kg/m²)	23.18 ± 3.16	22.47 ± 3.09	21.69 ± 3.33	22.50 ± 3.11	1.750	0.158
ECOG Performance Status, n (%))					5.060	0.483
0	6(9.4)	2(5.4)	3(8.3)	2(4.0)		
1	57(89.1)	35(94.6)	31(86.1)	48(96.0)		
2	1(1.6)	0(0.0)	2(5.6)	0(0.0)		
Tumor Type, n (%)					19.360	0.198
Gastrointestinal malignancies	19 (29.7)	15 (40.5)	14 (38.9)	17 (34.0)		
Gynecological malignancies	20 (31.3)	10 (27.0)	9 (25.0)	26 (52.0)		
Lung cancer	18 (28.1)	5 (13.5)	9 (25.0)	4 (8.0)		
Breast cancer	2 (3.1)	1 (2.7)	1 (2.8)	1 (2.0)		
Urinary system malignancies	2 (3.1)	4 (10.8)	2 (5.6)	1 (2.0)		
Other	3 (4.7)	2 (5.4)	1 (2.8)	1 (2.0)		
Clinical Stage, n (%)					11.323	0.228
I	1(1.6)	1(2.7)	1(2.8)	7(14.0)		
II	6(9.4)	4(10.8)	5(13.9)	4(8.0)		
III	8(12.5)	5(13.5)	8(22.2)	9(18.0)		
IV	49(76.6)	27(73.0)	22(61.1)	30(60.0)		
Hepatic Metastasis, n (%)	8(12.5)	11(29.7)	9(25.0)	12(24.0)	5.020	0.170
Bone Metastasis, n (%)	11(17.2)	3(8.1)	5(13.9)	2(4.0)	5.496	0.117
Chemotherapy Cycles, n (%)					5.603	0.133
<5 cycles	28(43.8)	17(45.9)	16(44.4)	32(64.0)		
>5 cycles	36(56.3)	20(54.1)	20(55.6)	18(36.0)		
Concurrent Radiotherapy, n (%)	8(12.5)	1(2.7)	4(11.1)	8(16.0)	4.169	0.239
Concurrent Targeted Therapy, n (%)	8(12.5)	4(10.8)	6(16.7)	7(14.0)	0.688	0.882

### Treatment efficacy rate

The combination therapy group, consisting of hetrombopag and rhTPO, exhibited a significantly higher treatment efficacy rate compared to the monotherapy groups, with an efficacy rate of 94.4% as opposed to 70.3% for both the rhTPO and hetrombopag monotherapy groups, and 66.0% for the rhIL-11 monotherapy group (P<0.05 for all comparisons). Importantly, no statistically significant differences were detected between the efficacy rates of hetrombopag monotherapy and either rhTPO monotherapy (P = 0.996) or rhIL-11 monotherapy (P = 0.673) (refer to **Tables 2**, **3**).

**Table 2:** Comparison of treatment efficacy rates across different regimens.

Primary outcome measure - 1	rhTPO Group (n=64)	Hetrombopag group (n=37)	Combination group (n=36)	rhIL-11 Group (n=50)	χ²	P-value
Number of effective cases (n, %)	45(70.3)	26(70.3)	34(94.4)	33(66.0)	10.149	0.017*

An asterisk (*) indicates statistically significant differences across all four treatment groups (P < 0.05*).

**Table 3:** Comparison of treatment efficacy rates between two regimens.

Comparison Groups	χ²	P-value	OR (95%*CI*)
rhTPO Group vs. Hetrombopag Group	0.000	0.996	1.002 (0.413,2.430)
rhTPO Group vs. Combination Group	8.088	0.004*	0.139 (0.030,0.639)
rhTPO Group vs. rhIL-11 Group	0.242	0.623	1.220 (0.552,2.698)
Hetrombopag Group vs. Combination Group	7.285	0.007*	0.139 (0.028,0.682)
Hetrombopag Group vs. rhIL-11 Group	0.178	0.673	1.218 (0.487,3.044)
rhIL-11 Group vs. Combination Group	9.839	0.002*	8.758 (1.875,40.910)

Note: An asterisk (*) indicates that the difference between the two groups was statistically significant, with P<0.05*.

### Platelet count absolute values before and after treatment

**Figure 1:**
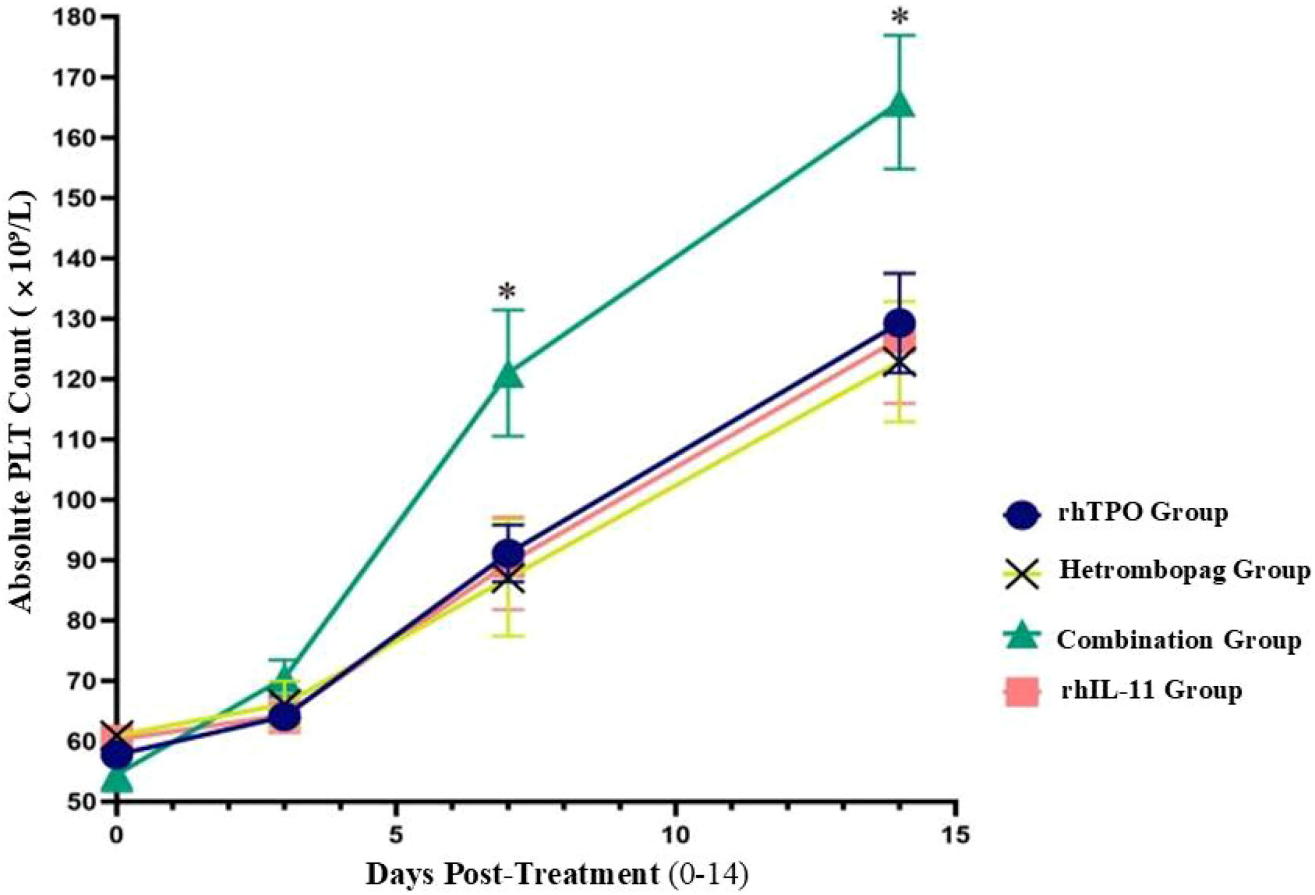
Comparison of absolute platelet counts (PLT, × 10^9^/L) across four treatment regiments at pre-treatment and Days 3, 7, and 14 post-treatment.

On Day (before treatment) and Day 3 post-treatment, there were no significant differences in platelet counts (PLT) among the four groups (P>0.05). However, by Days 7 and 14 post-treatment, the combination therapy group demonstrated significantly elevated PLT levels compared to the other three groups: the rhTPO group (P = 0.009, 0.008), the hetrombopag group (P = 0.017, 0.004), and the rhIL-11 group (P = 0.015, 0.011). The hetrombopag monotherapy group displayed efficacy comparable to both the rhTPO group (P = 0.704, 0.622) and the rhIL-11 group (P = 0.845, 0.796) (refer to **Tables 4**, **5**, **Figure 1**).

**Table 4:** Comparison of absolute platelet counts (PLT) before and after treatment.

Primary outcome measure-2	rhTPO Group (n=64)	Hetrombopag group (n=37)	Combination group (n=36)	rhIL-11 Group (n=50)	χ²	P-value
Pre-treatment PLT (×10^9^/L)	57.80 ± 1.67	60.95 ± 1.86	54.53 ± 2.79	60.30 ± 1.70	4.775	0.189
Day 3 PLT (×10^9^/L)	64.09 ± 1.76	66.17 ± 3.82	70.52 ± 2.95	64.33 ± 2.88	10.283	0.290
Day 7 PLT (×10^9^/L)	91.12 ± 4.73	87.04 ± 9.63	121.00 ± 10.46	89.45 ± 7.69	3.745	0.048*
Day 14 PLT (×10^9^/L)	129.28 ± 8.27	122.88 ± 10.03	165.84 ± 11.08	126.67 ± 10.70	7.665	0.016*
Pa	<0.001^#^	0.149	<0.001^#^	0.131		
*P^b^*	<0.001^#^	0.008^#^	<0.001^#^	<0.001^#^		
*P^c^*	<0.001^#^	<0.001^#^	<0.001^#^	<0.001^#^		

An asterisk (*) indicates statistically significant differences among the four treatment groups (P< 0.05*); (#) indicates statistically significant differences within group comparisons across time points (P < 0.05); Pa: Comparison between pre-treatment and post-treatment Day 3 PLT absolute values; Pb: Comparison between pre-treatment and post-treatment Day 7 PLT absolute values; Pc: Comparison between pre-treatment and post-treatment Day 14 PLT absolute values.

**Table 5:** Comparison of absolute platelet counts (PLT) between two regimens on days 7 and 14 post-treatment.

Time points	rhTPO vs. Hetrombopag	rhTPO vs. Combination	rhTPO vs. rhIL-11	Hetrombopag vs. Combination	Hetrombopag vs. rhIL-11	Combination vs. rhIL-11
Day 7	χ²=0.145, P = 0.704	χ²=6.776, P = 0.009*	χ²=0.034, P = 0.853	χ²=5.707, P = 0.017*	χ²=0.038, P = 0.845	χ²=5.908, P = 0.015*
Day 14	χ²=0.243, P = 0.622	χ²=6.994, P = 0.008*	χ²=0.037, P = 0.847	χ²=8.270, P = 0.004*	χ²=0.067, P = 0.796	χ²=6.471, P = 0.011*

An asterisk (*) indicates that the difference between the two groups was statistically significant, with P<0.05*.

### Time-to-platelet-recovery and platelet count difference (ΔPLT)

The median time to platelet recovery, defined as the first day on which the platelet count (PLT) was ≥100×10^9^/L, was significantly shorter in the combination therapy group, with a mean of 6.69 ± 2.21. This duration was notably less than that observed in the rhTPO group (9.12 ± 2.66, P = 0.008), the hetrombopag group (9.33 ± 3.24, P = 0.028), and the rhIL-11 group (9.25 ± 2.86, P = 0.014). No statistically significant differences were identified between the hetrombopag monotherapy group and either the rhTPO group (P = 0.836) or the rhIL-11 group (P = 0.941). Additionally, the combination therapy group exhibited a significantly greater ΔPLT, defined as the difference between pre-treatment and post-treatment platelet counts, compared to the other three groups: P = 0.005 (vs. rhTPO), P = 0.008 (vs. hetrombopag), and P = 0.002 (vs. rhIL-11). However, no significant differences in ΔPLT were found between the hetrombopag monotherapy group and either the rhTPO group (P = 0.860) or the rhIL-11 group (P = 0.825) (refer to **Tables 6**, **7**).

**Table 6:** Comparison of platelet recovery time and ΔPLT across regimens.

Primary outcome measure-3	rhTPO Group (n=64)	Hetrombopag group (n=37)	Combination group (n=36)	rhIL-11 Group (n=50)	F/H Value	P-value
Median Time-to-Platelet-Recovery (d)	9.12 ± 2.66	9.33 ± 3.24	6.69 ± 2.21	9.25 ± 2.86	3.029	0.035*
ΔPLT (×10^9^/L)	50.50 (29.50, 104.50)	56.00 (30.00, 90.00)	98.00 (58.50, 150.00)	50.00 (19.25, 117.00)	11.501	0.009*

An asterisk (*) indicates statistically significant differences across all four treatment groups (P < 0.05*).

**Table 7:** Pairwise Comparison of platelet recovery time and ΔPLT between regimens.

Primary outcome measure-3	rhTPO vs. Hetrombopag	rhTPO vs. Combination	rhTPO vs. rhIL-11	Hetrombopag vs. Combination	Hetrombopag vs. rhIL-11	Combination vs. rhIL-11
Median Time-to-Platelet-Recovery P-value	P=0.836	P=0.008*	P=0.876	P=0.028*	P=0.941	P=0.014*
Mean Difference (95%*CI*)	-0.212 (-2.326,1.902)	2.429 (0.751,4.106)	-0.129 (-1.798,1.540)	2.641 (0.224,5.058)	0.083 (-2.502,2.669)	-2.558 (-4.546,-0.570)
ΔPLT P-value	P=0.860	P=0.005*	P=0.655	P=0.008*	P=0.825	P=0.002*

Note: An asterisk (*) indicates that the difference between the two groups was statistically significant, with P<0.05*.

### Platelet transfusion rates

A total of six patients underwent platelet transfusions. The rates of platelet transfusion did not show significant variation across the four treatment groups (P = 0.842) (refer to **Table 8**).

**Table 8:** Comparison of platelet transfusion rates among treatment groups.

Secondary 0utcome measures-1	rhTPO Group(n=64)	Hetrombopag group (n=37)	Combination group (n=36)	rhIL-11 Group(n=50)	χ²	P-value
Platelet Transfusion Rate, n (%)	2(3.1)	2(5.4)	1(2.8)	1(2.0)	0.833	0.842

### Incidence of adverse reactions

A total of 32 patients experienced adverse reactions during treatment, with the most prevalent being transaminase elevation (7.8%-18.0%) and hyperbilirubinemia (2.8%-5.4%). No statistically significant differences were observed among the treatment groups (P > 0.05). Notably, no severe adverse reactions, such as thromboembolism, cataracts, or QT/QTc interval prolongation, were reported in any patient (refer to **Table 9**).

**Table 9:** Comparison of adverse reaction incidence rates among treatment groups.

Secondary 0utcome measures-2	rhtpo Group (n=64)	Hetrombopag group (n=37)	Combination group (n=36)	rhil-11 group (n=50)	χ²	P-value
transaminase elevation, n (%)	5(7.8)	5(13.5)	4(11.1)	9(18.0)	2.814	0.421
hyperbilirubinemia, n (%)	3(4.7)	2(5.4)	1(2.8)	2(4.0)	0.557	1.000
fatigue, n (%)	1(1.6)	0(0.00)	0(0.00)	1(2.0)	1.484	1.000
headache, n (%)	1(1.6)	0(0.00)	0(0.00)	1(2.0)	1.484	1.000
total adverse reactions, n (%)	10(15.6)	5(13.5)	5(13.9)	12(24.0)	2.374	0.499

## Discussion

Thrombocytopenia is a prevalent complication among cancer patients, primarily resulting from antitumor therapies such as chemotherapy. Its incidence has been reported to be as high as 12.8% in patients undergoing chemotherapy for solid tumors, with 6.4% experiencing grade 2, 4.2% experiencing grade 3, and 1.9% experiencing grade 4 thrombocytopenia (3, 8). As of now, six thrombopoiesis-stimulating agents have received global approval for clinical application: recombinant human thrombopoietin (rhTPO), romiplostim, eltrombopag, hetrombopag, avatrombopag, and lusutrombopag. Recombinant human thrombopoietin (rhTPO) is a full-length glycosylated protein produced and purified from Chinese hamster ovary (CHO) cells using recombinant DNA technology, exhibiting 99% homology with natural thrombopoietin (TPO). This protein interacts with the extracellular domain of the TPO receptor, inducing conformational changes that activate three critical signaling pathways: JAK/STAT, RAS/MAPK, and PI3K/AKT. Through these pathways, rhTPO facilitates the differentiation and maturation of multipotent hematopoietic stem cells, megakaryocyte progenitor cells, and polyploid megakaryocytes, ultimately enhancing platelet production. In contrast, orally administered small-molecule non-peptide TPO receptor agonists (TPO-RAs) interact with the transmembrane domain of the TPO receptor, initiating signaling cascades that promote the proliferation and differentiation of myeloid progenitors and megakaryocytes. Thrombopoietin receptor agonists (TPO-RAs) do not compete with endogenous thrombopoietin (TPO) molecules for receptor binding sites and demonstrate additive effects when combined with endogenous TPO (9). This mechanistic profile indicates that combination therapy could substantially enhance the efficiency of platelet production through synergistic multi-target interactions.

Eltrombopag, the first orally administered TPO-RA to receive global clinical approval, has been authorized by both the Food and Drug Administration (FDA) and the European Medicines Agency (EMA) for the treatment of chronic immune thrombocytopenia (ITP), hepatitis C virus (HCV)-associated thrombocytopenia, and newly diagnosed or refractory severe aplastic anemia (SAA). In patients undergoing gemcitabine-platinum chemotherapy, eltrombopag administration resulted in a significant improvement in platelet recovery kinetics compared to placebo, reducing the median platelet recovery time by 6.7 days (8.1 days versus 14.8 days, respectively) (10). Notably, among patients who developed grade 3/4 CIT, eltrombopag achieved a 75% response rate within 7 days. However, it is important to note that eltrombopag is associated with an increased risk of hepatotoxicity, primarily evidenced by elevated serum transaminases (ALT/AST) and bilirubin levels (11, 12). Hetrombopag, an orally administered TPO-RA developed in China, has undergone structural optimization through three modifications compared to eltrombopag: (1) the incorporation of hydrophobic moieties to increase lipophilicity, (2) the optimization of metal chelation domains, and (3) the modification of acidic fragments to enhance bioactivity (13). These structural improvements have led to a 60% reduction in the incidence of grade≥3 hepatic enzyme elevation compared to eltrombopag, while maintaining equivalent efficacy, as evidenced by a median platelet recovery time of 7.5 days, at only one-tenth of the daily dose (6). This pharmacological advancement offers a superior risk-benefit profile in the treatment of refractory immune thrombocytopenia (ITP) and severe aplastic anemia (SAA), providing a clinically safer therapeutic alternative.

Avatrombopag is an advanced oral thrombopoietin receptor agonist (TPO-RA) characterized by a molecular structure devoid of metal ion chelating groups, thereby eliminating dietary restrictions and allowing administration with meals. Variables such as age, body weight, gender, race, hepatic impairment, and mild to moderate renal impairment do not exert clinically significant effects on its pharmacokinetics. A global, multicenter, randomized, placebo-controlled phase III clinical trial assessing its efficacy and safety in chemotherapy-induced thrombocytopenia (CIT) revealed no statistically significant differences between the avatrombopag and placebo groups regarding the achievement of primary study endpoints or the incidence of adverse events (14). Conversely, a phase III, multicenter, open-label, single-arm clinical trial conducted in China reported a cumulative response rate of 70.3% at 4 weeks following avatrombopag treatment (60 mg/day for 5–10 days), with 56.8% of patients achieving platelet counts ≥100×10^9^/L and a median platelet recovery time of (10.2 ± 6.4) days (15).Romiplostim, a recombinant Fc-peptide fusion protein engineered through DNA technology, is the first thrombopoietin (TPO) peptidomimetic agent to receive global approval. Research on the use of romiplostim in the management of chemotherapy-induced thrombocytopenia (CIT) has demonstrated its ability to swiftly increase platelet counts (16), although it may also elevate the risk of venous thrombosis in patients with solid tumors (17).

In terms of efficacy, this retrospective cohort study demonstrated that the combination of hetrombopag and recombinant human thrombopoietin (rhTPO) was more effective in achieving faster platelet count (PLT) recovery at days 7 and 14 post-treatment compared to rhTPO monotherapy, hetrombopag monotherapy, or rhIL-11 treatment. This combination significantly reduced the time required for PLT recovery to ≥100 × 10^9^/L (P < 0.05), which is of critical clinical importance in reducing bleeding risks and minimizing treatment delays. However, the presence of a hydrazide structure in hetrombopag, which readily chelates metal cations, may interfere with cation absorption. Additionally, the stringent dosing schedule and gastrointestinal requirements of hetrombopag may limit its use in patients with concurrent gastrointestinal disorders or those requiring polypharmacy. Although two studies—one involving patients with grade 3/4 CIT (n = 28) and another retrospective analysis of CIT in lymphoma and myeloma patients (n = 60)—reported favorable outcomes with hetrombopag monotherapy or combination therapy, the small sample sizes may reduce statistical power, potentially compromising the precision of efficacy assessment (18, 19).

In terms of safety, while no significant differences in the overall rates of adverse events were detected among the four treatment regimens examined in this study, continued vigilance is necessary for the potential elevation of transaminases (particularly alanine aminotransferase [ALT] and aspartate aminotransferase [AST]), thrombocytosis, and hyperbilirubinemia during hetrombopag treatment for CIT (20). It is recommended that clinicians conduct regular monitoring of hepatic function, especially in patients with pre-existing hepatic impairment or a history of abnormal liver enzyme levels.

### Limitations of this study

The retrospective nature of the analysis depended on the accuracy and completeness of medical record data, which may introduce information bias or result in missing data. The relatively small sample size may restrict the generalizability of the findings. The study cohort comprised patients with solid organ tumors, characterized by heterogeneity in tumor types and chemotherapy regimens, which may affect the accuracy of the assessment. Although four treatment regimens were compared, the study lacked long-term follow-up data to evaluate the durability of treatment effects and delayed safety profiles. Variability in baseline comorbidities, the intensity of chemotherapy, and the severity of CIT among patients may confound the outcomes. Additionally, the study primarily concentrated on platelet recovery time, neglecting critical clinical endpoints such as the chemotherapy cycle completion rate (CCRT) and the reduction in bleeding events.

### Future research directions clinical trial expansion

1. Multicenter, prospective studies are necessary to validate the efficacy of combination therapies in managing severe CIT (platelet count <25 × 10^9^/L) and in high-risk populations, such as elderly patients and those with comorbid liver diseases. Extended follow-up periods, such as three months post-chemotherapy, are essential for evaluating long-term safety profiles.
2. Biomarker Development: A subset of patients demonstrates inadequate responses to combination therapies, possibly due to aberrant thrombopoietin (TPO) receptor signaling pathways or suppressed bone marrow microenvironments. The development of novel biomarkers is crucial to guide personalized treatment strategies.
3. Dose Optimization: The variability in synergistic effects between different thrombopoietin receptor agonists (TPO-RA) and recombinant human thrombopoietin (rhTPO) necessitates large-scale studies to determine optimal dose combinations.
4. Next-Generation TPO-RA Development: In addressing current limitations, the development of next-generation TPO-RAs should prioritize high selectivity, low immunogenicity, and oral bioavailability. Strategies may encompass the utilization of nanoparticle-based drug delivery systems to enhance targeting capabilities or the development of bispecific antibody designs to achieve dual mechanisms of action.

## Conclusion

In conclusion, hetrombopag is currently approved for the treatment of immune thrombocytopenia (ITP) and severe aplastic anemia (SAA). Its ability to facilitate rapid platelet recovery and its manageable safety profile in CIT treatment, particularly when used in conjunction with recombinant human thrombopoietin (rhTPO), highlight its potential as a promising therapeutic option. Future progress in structural biology, translational research, and the integration of real-world data are crucial to overcoming existing limitations and establishing safer, more effective platelet management strategies for oncology patients.

## Data Availability

The raw data supporting the conclusions of this article will be made available by the authors, without undue reservation.
